# (*R*)-*N*-(3-Meth­oxy­phen­yl)-*tert*-butane­sulfinamide

**DOI:** 10.1107/S1600536812006496

**Published:** 2012-02-17

**Authors:** Xiaofei Sun, Chuan Dai, Xingzhao Tu, Wenguo Wang, Qingle Zeng

**Affiliations:** a Institute of Green Catalysis and Synthesis, College of Materials and Chemistry and Chemical Engineering, Chengdu University of Technology, Chengdu 610059, People’s Republic of China; b Fujian Institute of Research on the Structure of Matter, Chinese Academy of Sciences, Fuzhou 350002, People’s Republic of China

## Abstract

The title compound, C_11_H_17_NO_2_S, was obtained by the reaction of (*R*)-*tert*-butane­sulfinamide with 3-meth­oxy­phenyl bromide in toluene. In the crystal, mol­ecules inter­act head-to-tail through N—H⋯O and C—H⋯O hydrogen bonds, forming one-dimensional chains parallel to the *a* axis.

## Related literature

For the structure of the racemic title compound, see: Datta *et al.* (2010 ▶). For the structures of related *N*-aryl­alkanesulfinamides, see: Datta *et al.* (2008 ▶, 2009*a* ▶,*b* ▶). For the structures of related *N*-alkyl­alkanesulfinamides, see: Sato *et al.* (1975 ▶); Schuckmann *et al.* (1978 ▶); Ferreira *et al.* (2005 ▶).
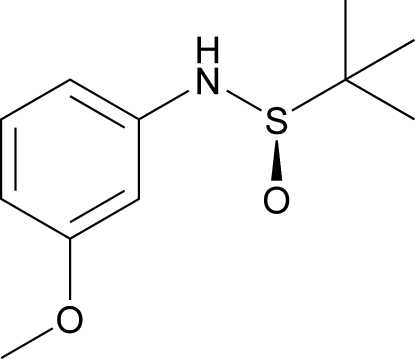


## Experimental

### 

#### Crystal data

C_11_H_17_NO_2_S
*M* *_r_* = 227.33
Orthorhombic, 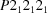

*a* = 7.4418 (9) Å
*b* = 9.7027 (12) Å
*c* = 16.862 (2) Å
*V* = 1217.5 (3) Å^3^
*Z* = 4
Mo *K*α radiation
μ = 0.25 mm^−1^
*T* = 293 K
0.30 × 0.20 × 0.20 mm

#### Data collection

Oxford Diffraction Xcalibur Eos diffractometer
Absorption correction: multi-scan (*CrysAlis PRO*; Oxford Diffraction, 2010 ▶) *T*_min_ = 0.990, *T*_max_ = 1.0
7010 measured reflections
2481 independent reflections
2062 reflections with *I* > 2σ(*I*)
*R*_int_ = 0.031

#### Refinement

*R*[*F* ^2^ > 2σ(*F* ^2^)] = 0.043
*wR*(*F* ^2^) = 0.092
*S* = 1.10
2481 reflections
204 parameters
All H-atom parameters refined
Δρ_max_ = 0.22 e Å^−3^
Δρ_min_ = −0.29 e Å^−3^
Absolute structure: Flack (1983 ▶), 1029 Friedel pairs
Flack parameter: −0.02 (9)

### 

Data collection: *CrysAlis PRO* (Oxford Diffraction, 2010 ▶); cell refinement: *CrysAlis PRO*; data reduction: *CrysAlis PRO*; program(s) used to solve structure: *SHELXS97* (Sheldrick, 2008 ▶); program(s) used to refine structure: *SHELXL97* (Sheldrick, 2008 ▶); molecular graphics: *OLEX2* (Dolomanov *et al.*, 2009 ▶); software used to prepare material for publication: *OLEX2*.

## Supplementary Material

Crystal structure: contains datablock(s) I, global. DOI: 10.1107/S1600536812006496/rz2711sup1.cif

Structure factors: contains datablock(s) I. DOI: 10.1107/S1600536812006496/rz2711Isup4.hkl

Supplementary material file. DOI: 10.1107/S1600536812006496/rz2711Isup3.cml

Additional supplementary materials:  crystallographic information; 3D view; checkCIF report

## Figures and Tables

**Table 1:** Hydrogen-bond geometry (Å, °)

*D*—H⋯*A*	*D*—H	H⋯*A*	*D*⋯*A*	*D*—H⋯*A*
N1—H1⋯O2^i^	0.80 (2)	2.28 (3)	3.031 (3)	157 (2)
C10—H10*A*⋯O2^i^	1.02 (3)	2.47 (3)	3.487 (4)	171 (2)

Symmetry code: (i) 
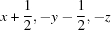
.

**Fig. 1.**
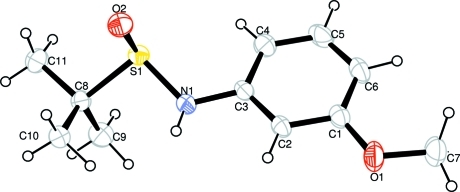
The molecular structure of the title compound with 50% probability displacement ellipsoids for non-H atoms.

**Fig. 2.**
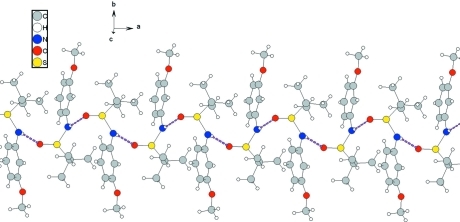
The chain structure of the title compound formed by intermolecular N—H···O hydrogen bonds (dashed lines).

## supplementary crystallographic information

### Comment

Sulfinamides, especially chiral sulfinamides, are an important class of organic compounds in modern organic chemistry, and a great number of such compounds have been synthesized. In our study on sulfinamides, we have prepared the title compound and report its crystal structure herein.

In the molecule of the title compound (Fig. 1), the N–C_aryl_ bond length [1.416 (3) Å] is quite similar to that found in the racemic 3-MeO-N-phenyl-tert-butanesulfinamide (1.418 (2) Å; Datta *et al.*, 2010), and could be compared with those reported for 4-MeO-N-phenyl-tert-butanesulfinamide (1.4225 (14) Å; Datta *et al.*, 2009*a*), N-phenyl-tert-butanesulfinamide (1.4083 (12) Å; Datta *et al.*, 2009*b*) and other N-alkylalkanesulfinamides (1.470–1.530 Å; Sato *et al.*, 1975; Schuckmann *et al.*, 1978; Ferreira *et al.*, 2005). The crystal packing shows an intermolecular interaction through N-H···O=S hydrogen bond, forming a chain structure parallel to the *a* axis (Fig. 2; Table 1). In addition, the chain is enforced by an intermolecular C-H···O=S hydrogen bond as observed in the crystal packing of N-phenyladamantane-1-sulfinamide (Datta *et al.*, 2008).

### Experimental

A oven-dried ground test tube, which was equipped with a magnetic stir bar and fitted with a rubber septum, was charged with (R)-tert-butanesulfinamide (0.121 g, 1.0 mmol), Pd_2_(dba)_3_ (0.018 g, 0.02 mmol; dba is dibenzylideneacetone), 2-di-tert-butylphosphino-2',4',6'-triisopropylbiphenyl (0.0212 g, 0.05 mmol) and NaOH (0.08 g, 2 mmol). The vessel was evacuated and backfilled with argon three times, then 3-methoxyphenyl bromide (1.3 mmol), toluene (10 ml) and degassed water (0.3 mL) were added via syringe. The solution was stirred at 90°C for 20 h. The reaction mixture was then cooled to room temperature, quenched by water, and extracted with ethyl acetate (20 mL) for twice. The organic layer was combined, and dried over anhydrous sodium sulfate and filtrated. The filterate was condensed under vacuum. The residual was purified with silica gel column chromatography with a solution of petroleum ether and ethyl acetate (5:1 *v*:*v*) as eluent. A test tube containing the eluate was covered with a piece of filter paper and placed motionless at room temperature, and a single crystal was cultured in the bottom of the test tube. Yield: 0.186 g, 82%. Spectroscopic analysis: ESI-MS (negative mode), m/z = 226 [M-H]-. FTIR (KBr) (cm^-1^): 3456, 3273, 3112, 3076, 2966, 1584, 1519, 1246, 1186, 1113, 1068, 875, 795, 751. ^1^H NMR (300 MHz, CDCl_3_), δ (ppm): 7.16 (t, J = 8.2 Hz, 1H), 5.60-5.55 (m, 3H), 5.34 (s, 1H), 3.78(s, 3H), 1.33 (s, 9H). ^13^C NMR (300 MHz, CD_3_OD), δ (ppm): 160.2, 143.5, 129.8, 110.1, 107.9, 103.5, 56.3, 54.9, 22.3. [α]D = -2.6 (c 0.05, ethyl acetate).

### Refinement

All H atoms were located in a difference Fourier map and refined freely (N—H = 0.80 (2) Å; C—H = 0.90 (3)–1.03 (3) Å). The absolute configuration was assigned by reference to the unchanging chiral centre in the synthetic procedure.

### Crystal data

**Table:**

C_11_H_17_NO_2_S	*D*_x_ = 1.246 Mg m^−^^3^
*M**_r_* = 227.33	Melting point: 375 K
Orthorhombic, *P*2_1_2_1_2_1_	Mo *K*α radiation, λ = 0.7107 Å
Hall symbol: P 2ac 2ab	Cell parameters from 2411 reflections
*a* = 7.4418 (9) Å	θ = 3.0–29.1°
*b* = 9.7027 (12) Å	µ = 0.25 mm^−^^1^
*c* = 16.862 (2) Å	*T* = 293 K
*V* = 1217.5 (3) Å^3^	Block, colourless
*Z* = 4	0.30 × 0.20 × 0.20 mm
*F*(000) = 488	

### Data collection

**Table:**

Oxford Diffraction Xcalibur Eos diffractometer	2481 independent reflections
Radiation source: fine-focus sealed tube	2062 reflections with *I* > 2σ(*I*)
Graphite monochromator	*R*_int_ = 0.031
Detector resolution: 16.0874 pixels mm^-1^	θ_max_ = 26.4°, θ_min_ = 3.0°
ω scans	*h* = −9→6
Absorption correction: multi-scan (*CrysAlis PRO*; Oxford Diffraction, 2010)	*k* = −12→12
*T*_min_ = 0.990, *T*_max_ = 1.0	*l* = −18→21
7010 measured reflections	

### Refinement

**Table:**

Refinement on *F*^2^	Secondary atom site location: difference Fourier map
Least-squares matrix: full	Hydrogen site location: inferred from neighbouring sites
*R*[*F*^2^ > 2σ(*F*^2^)] = 0.043	All H-atom parameters refined
*wR*(*F*^2^) = 0.092	*w* = 1/[σ^2^(*F*_o_^2^) + (0.0403*P*)^2^] where *P* = (*F*_o_^2^ + 2*F*_c_^2^)/3
*S* = 1.10	(Δ/σ)_max_ < 0.001
2481 reflections	Δρ_max_ = 0.22 e Å^−^^3^
204 parameters	Δρ_min_ = −0.29 e Å^−^^3^
0 restraints	Absolute structure: Flack (1983), 1029 Friedel pairs
Primary atom site location: structure-invariant direct methods	Flack parameter: −0.02 (9)

### Special details

**Table:**

Geometry. All e.s.d.'s (except the e.s.d. in the dihedral angle between two l.s. planes) are estimated using the full covariance matrix. The cell e.s.d.'s are taken into account individually in the estimation of e.s.d.'s in distances, angles and torsion angles; correlations between e.s.d.'s in cell parameters are only used when they are defined by crystal symmetry. An approximate (isotropic) treatment of cell e.s.d.'s is used for estimating e.s.d.'s involving l.s. planes.
Refinement. Refinement of *F*^2^ against ALL reflections. The weighted *R*-factor *wR* and goodness of fit *S* are based on *F*^2^, conventional *R*-factors *R* are based on *F*, with *F* set to zero for negative *F*^2^. The threshold expression of *F*^2^ > σ(*F*^2^) is used only for calculating *R*-factors(gt) *etc*. and is not relevant to the choice of reflections for refinement. *R*-factors based on *F*^2^ are statistically about twice as large as those based on *F*, and *R*- factors based on ALL data will be even larger.

### Fractional atomic coordinates and isotropic or equivalent isotropic displacement parameters (Å^2^)

**Table:**

	*x*	*y*	*z*	*U*_iso_*/*U*_eq_	
S1	−0.84124 (9)	−0.41552 (6)	−0.07783 (3)	0.04091 (18)	
O1	−0.6725 (4)	0.18196 (19)	−0.18439 (11)	0.0691 (6)	
O2	−1.0171 (2)	−0.39294 (19)	−0.03812 (10)	0.0543 (5)	
N1	−0.7259 (3)	−0.2699 (2)	−0.08452 (13)	0.0464 (5)	
C1	−0.7230 (3)	0.0498 (3)	−0.20153 (15)	0.0468 (7)	
C2	−0.7075 (3)	−0.0421 (3)	−0.13905 (15)	0.0416 (6)	
C3	−0.7532 (3)	−0.1789 (3)	−0.14907 (14)	0.0390 (6)	
C4	−0.8154 (4)	−0.2233 (3)	−0.22198 (15)	0.0540 (7)	
C5	−0.8331 (5)	−0.1298 (3)	−0.28284 (17)	0.0627 (8)	
C6	−0.7857 (4)	0.0065 (3)	−0.27436 (17)	0.0561 (8)	
C7	−0.6704 (6)	0.2798 (4)	−0.2476 (2)	0.0730 (10)	
C8	−0.6971 (3)	−0.5045 (3)	−0.00507 (14)	0.0423 (6)	
C9	−0.5083 (4)	−0.5131 (4)	−0.0392 (2)	0.0621 (8)	
C10	−0.7023 (5)	−0.4311 (3)	0.07445 (17)	0.0527 (7)	
C11	−0.7817 (6)	−0.6475 (3)	0.0011 (2)	0.0618 (9)	
H9C	−0.509 (4)	−0.550 (3)	−0.0879 (18)	0.060 (9)*	
H2	−0.667 (3)	−0.012 (2)	−0.0913 (14)	0.041 (6)*	
H10A	−0.641 (4)	−0.337 (3)	0.0697 (16)	0.074 (9)*	
H7B	−0.593 (4)	0.247 (3)	−0.2904 (19)	0.071 (10)*	
H9B	−0.438 (5)	−0.567 (4)	−0.005 (2)	0.113 (14)*	
H9A	−0.439 (4)	−0.423 (3)	−0.0417 (16)	0.065 (9)*	
H5	−0.878 (4)	−0.161 (3)	−0.3324 (17)	0.060 (8)*	
H6	−0.807 (4)	0.066 (3)	−0.3158 (15)	0.056 (8)*	
H1	−0.692 (4)	−0.237 (2)	−0.0438 (14)	0.039 (7)*	
H4	−0.845 (4)	−0.312 (3)	−0.2268 (15)	0.056 (8)*	
H11C	−0.913 (5)	−0.641 (3)	0.0169 (18)	0.069 (10)*	
H10C	−0.819 (4)	−0.425 (3)	0.0947 (17)	0.077 (10)*	
H11A	−0.709 (4)	−0.705 (3)	0.0376 (17)	0.071 (9)*	
H10B	−0.643 (4)	−0.481 (3)	0.1119 (16)	0.061 (8)*	
H7A	−0.620 (5)	0.361 (4)	−0.224 (2)	0.103 (14)*	
H7C	−0.793 (5)	0.285 (4)	−0.2714 (18)	0.095 (13)*	
H11B	−0.775 (4)	−0.695 (3)	−0.0537 (18)	0.075 (9)*	

### Atomic displacement parameters (Å^2^)

**Table:**

	*U*^11^	*U*^22^	*U*^33^	*U*^12^	*U*^13^	*U*^23^
S1	0.0392 (3)	0.0430 (3)	0.0405 (3)	−0.0007 (3)	−0.0001 (3)	−0.0027 (3)
O1	0.0977 (18)	0.0545 (11)	0.0550 (11)	−0.0043 (13)	−0.0024 (13)	0.0189 (9)
O2	0.0394 (10)	0.0641 (12)	0.0594 (12)	0.0039 (10)	0.0060 (9)	0.0031 (9)
N1	0.0555 (14)	0.0464 (12)	0.0374 (12)	−0.0082 (10)	−0.0103 (12)	0.0041 (10)
C1	0.0424 (16)	0.0568 (16)	0.0414 (13)	0.0049 (12)	0.0027 (13)	0.0091 (11)
C2	0.0401 (15)	0.0520 (15)	0.0326 (13)	0.0047 (11)	0.0005 (12)	0.0048 (11)
C3	0.0304 (14)	0.0538 (14)	0.0328 (12)	0.0025 (11)	0.0020 (12)	0.0059 (10)
C4	0.061 (2)	0.0594 (17)	0.0419 (15)	−0.0038 (16)	−0.0022 (14)	−0.0001 (13)
C5	0.067 (2)	0.088 (2)	0.0333 (14)	−0.0040 (19)	−0.0088 (17)	−0.0004 (13)
C6	0.0509 (18)	0.076 (2)	0.0412 (15)	0.0086 (16)	0.0015 (14)	0.0183 (14)
C7	0.075 (3)	0.075 (2)	0.068 (2)	−0.007 (2)	0.004 (2)	0.0300 (18)
C8	0.0428 (15)	0.0392 (12)	0.0448 (14)	0.0019 (12)	−0.0018 (12)	0.0021 (10)
C9	0.0529 (19)	0.065 (2)	0.068 (2)	0.0114 (18)	0.0010 (19)	−0.0014 (18)
C10	0.0557 (19)	0.0586 (17)	0.0439 (14)	−0.0086 (15)	−0.0047 (16)	0.0064 (14)
C11	0.072 (3)	0.0414 (16)	0.072 (2)	−0.0058 (15)	−0.007 (2)	0.0048 (15)

### Geometric parameters (Å, º)

**Table:**

S1—O2	1.4866 (18)	C6—H6	0.92 (3)
S1—N1	1.657 (2)	C7—H7B	0.98 (3)
S1—C8	1.844 (2)	C7—H7A	0.96 (4)
O1—C1	1.367 (3)	C7—H7C	1.00 (4)
O1—C7	1.428 (3)	C8—C9	1.521 (4)
N1—C3	1.416 (3)	C8—C10	1.519 (4)
N1—H1	0.80 (2)	C8—C11	1.527 (4)
C1—C2	1.385 (3)	C9—H9C	0.90 (3)
C1—C6	1.379 (4)	C9—H9B	0.93 (4)
C2—C3	1.381 (3)	C9—H9A	1.01 (3)
C2—H2	0.91 (2)	C10—H10A	1.02 (3)
C3—C4	1.382 (4)	C10—H10C	0.94 (3)
C4—C5	1.376 (4)	C10—H10B	0.91 (3)
C4—H4	0.90 (3)	C11—H11C	1.02 (3)
C5—C6	1.376 (4)	C11—H11A	0.99 (3)
C5—H5	0.95 (3)	C11—H11B	1.03 (3)
			
S1—N1—H1	116.3 (18)	C8—C9—H9C	111.4 (19)
O1—C1—C2	114.8 (2)	C8—C9—H9B	109 (2)
O1—C1—C6	124.5 (2)	C8—C9—H9A	115.9 (17)
O1—C7—H7B	109.7 (18)	C8—C10—H10A	109.8 (16)
O1—C7—H7A	104 (2)	C8—C10—H10C	112.1 (19)
O1—C7—H7C	109 (2)	C8—C10—H10B	110.3 (17)
O2—S1—N1	111.17 (12)	C8—C11—H11C	110.9 (17)
O2—S1—C8	106.35 (11)	C8—C11—H11A	109.0 (18)
N1—S1—C8	98.23 (12)	C8—C11—H11B	108.7 (17)
C1—O1—C7	118.0 (3)	C9—C8—S1	108.1 (2)
C1—C2—H2	119.6 (15)	C9—C8—C11	110.9 (3)
C1—C6—H6	122.8 (16)	C10—C8—S1	110.65 (19)
C2—C3—N1	118.0 (2)	C10—C8—C9	112.5 (3)
C2—C3—C4	119.4 (2)	C10—C8—C11	110.8 (2)
C3—N1—S1	120.62 (19)	C11—C8—S1	103.4 (2)
C3—N1—H1	117.4 (17)	H9C—C9—H9B	110 (3)
C3—C2—C1	120.3 (2)	H9C—C9—H9A	108 (3)
C3—C2—H2	120.1 (15)	H10A—C10—H10C	113 (3)
C3—C4—H4	117.5 (17)	H10A—C10—H10B	108 (2)
C4—C3—N1	122.5 (2)	H7B—C7—H7A	110 (3)
C4—C5—C6	122.1 (3)	H7B—C7—H7C	105 (3)
C4—C5—H5	118.5 (17)	H9B—C9—H9A	103 (3)
C5—C4—C3	119.4 (3)	H11C—C11—H11A	114 (3)
C5—C4—H4	123.1 (17)	H11C—C11—H11B	108 (3)
C5—C6—C1	118.2 (3)	H10C—C10—H10B	103 (2)
C5—C6—H6	118.8 (16)	H11A—C11—H11B	106 (2)
C6—C1—C2	120.6 (3)	H7A—C7—H7C	119 (3)
C6—C5—H5	119.4 (17)		

### Hydrogen-bond geometry (Å, º)

**Table:**

*D*—H···*A*	*D*—H	H···*A*	*D*···*A*	*D*—H···*A*
N1—H1···O2^i^	0.80 (2)	2.28 (3)	3.031 (3)	157 (2)
C10—H10*A*···O2^i^	1.02 (3)	2.47 (3)	3.487 (4)	171 (2)

Symmetry code: (i) *x*+1/2, −*y*−1/2, −*z*.
